# Impact of the 16^th ^International Conference on AIDS: can these conferences lead to policy change?

**DOI:** 10.1186/1742-4690-4-13

**Published:** 2007-02-08

**Authors:** Helene Gayle, Mark A Wainberg

**Affiliations:** 1 CARE USA, Atlanta 30303, GA, USA; 2 McGill University AIDS Centre, Montreal H3T 1E2, Canada

## Abstract

This Commentary reflects on the success of the XVI International Conference on AIDS, that was held in Toronto between August 13–18, 2006. Not only was the Conference judged to have been a scientific success, it will probably also be recognized over time as having had important political impact. It is vital that scientists and policy-makers continue to be able to interact at these meetings as part of global efforts to combat the HIV epidemic.

## Commentary

For one week in August 2006, more than 26,000 attendees from 170 countries participated in the sixteenth biennial International AIDS Conference, held in Toronto [1]. These conferences were initially started primarily as a way to exchange the most important ground-breaking scientific findings among the community of scientists and clinicians working on the pandemic. However, over the years, activism and advocacy have become equally important components of these meetings. Indeed, the International AIDS Conference held in Vancouver, 1996, with the theme "One World, One Hope" helped to build the momentum for activism focused on global treatment access that was the foundation of the World Health Organization 3 × 5 campaign and the commitment by the G8 countries to deliver universal access for HIV treatment and prevention. The theme in 2006 of "Time to Deliver" was a call for accountability and to honour our commitments to people and communities living with HIV.

As repeated throughout the conference, one example of a great gap in accountability was the South African government's policy towards the HIV epidemic. Remarkably, the past three months have now witnessed a veritable revolution in South African government leadership and policy related to the AIDS epidemic. This long overdue change includes official recognition that HIV is the cause of AIDS and a commitment to provide antiretroviral drugs (ARVs) to the millions of HIV-infected South Africans who will die unless these life-saving medications are made available. This reversal in government policy comes after more than six years of official disclaimers that ARVs provided benefit and denial by President Mbeki himself that HIV was the cause of AIDS. Organizers of the International AIDS Conference, held in Durban, South Africa in 2000, will never forget the shock of realization that they and the South African government were at cross- purposes on the most important public health issue of our day.

What has now prompted these welcome changes in South African policy and did the International AIDS Conference itself have a role?

First, credit must go to the relentless efforts of people within South Africa, including HIV/AIDS activists, clinicians, government officials, politicians, and journalists. No-one who attended the Durban conference of 2000 will ever forget the remarks of South African Supreme Court Justice Edwin Cameron, who declared that he was gay, HIV-infected, and the fortunate recipient of ARVs. From that time to the present, coalitions of activist groups led by Zackie Achmat and Mark Heywood of the Treatment Action Campaign (TAC), and members of the South African HIV Clinicians Society have been united in attempts to bring rational decision-making in AIDS government policy to their country.

In addition, it appears that the calls for accountability emanating from the International AIDS Conference in Toronto in August 2006 may also have made an important difference. The South African Minister of Health, Manto Tshabalala-Msimang, attended the Conference and remarkably stated that consumption of lemon juice, garlic, and beetroot, rather than use of ARVs, was the best way to combat the epidemic. These comments were promptly derided  as preposterous by numerous scientists and clinicians at the Conference, including ourselves. Celebrities, activists, and politicians in attendance, such as Bill Gates, Bill Clinton, Richard Gere, and Stephen Lewis all added their voice to concerns about the statements of the Minister and, more broadly, about the overall South African response,, and their comments were widely quoted in both the international and the South African press. Perhaps, this helped to develop a consensus within South Africa that Minister Manto was no longer an appropriate spokesperson on the topic of HIV/AIDS and was counterproductive to South Africa's international image and its response to the HIV epidemic. Soon after the conference, it was announced that her Deputy Minister, Nozizwe Madlala-Routledge, would now assume responsibility for the HIV/AIDS portfolio. Furthermore, Deputy-President Phumzile Mlambo-Ngcuka, rather than President Mbeki himself, would now be in charge of overall government policy on AIDS.  

These changes suggest that pressures within the African National Congress, the governing party of South Africa, must finally have become so intense as to force this turnaround. Doubtless, the timing is also related to multiple political and other factors that impacted on these decisions.

We may never know the reasons that Mbeki, one of the most respected African leaders, was captured by HIV denialism. What is clear, however, is that the confusion sown by his denials of HIV as the cause of AIDS may have resulted in the needless, additional infections of hundreds of thousands of his co-citizens.

It is impossible to know the role that the 2006 International AIDS Conference played in influencing South Africa's AIDS policy. However, the many voices, including those of internationally recognized figures, must surely have played some part in making patently obvious the increasingly negative impact that these policies were having on South Africa's image and on the lives of people with HIV in South Africa.

This is but one example of the powerful role these conferences have played over the years in shaping policy. The stories that journalists wrote and broadcast from the Conference raised HIV/AIDS awareness throughout the world, and probably did more to prevent new cases of HIV transmission than any single scientific presentation at the Conference, although, to be sure, the Conference included a large number of novel reports that point the way to improvements in the prevention and treatment of HIV disease.

This and the fact that the International AIDS Conference seems to have played a role in catalyzing government change in South Africa on the topic of AIDS is one example of the importance of continuing to organize this event on a semi-annual basis. The International AIDS Conference attracts more media attention than any medical conference in the world, and the AIDS community must take advantage of this for as long as HIV/AIDS remains the world's most important public health problem. We are grateful to have had the opportunity to work together with our colleagues at the International AIDS Society, at several international non-governmental organizations, and with member of the Toronto Local Host community toward the success of this major event.

## Competing interests

The author(s) declare that they have no competing interests.
